# Dissociating Arithmetic Operations in the Parietal Cortex Using 1 Hz Repetitive Transcranial Magnetic Stimulation: The Importance of Strategy Use

**DOI:** 10.3389/fnhum.2020.00271

**Published:** 2020-07-16

**Authors:** Shane Fresnoza, Monica Christova, Sieglinde Purgstaller, Margit Jehna, Karla Zaar, Markus Hoffermann, Kariem Mahdy Ali, Christof Körner, Eugen Gallasch, Gord von Campe, Anja Ischebeck

**Affiliations:** ^1^Institute of Psychology, University of Graz, Graz, Austria; ^2^BioTechMed, Graz, Austria; ^3^Otto Loewi Research Center, Physiology Section, Medical University of Graz, Graz, Austria; ^4^Department of Physiotherapy, University of Applied Sciences FH-Joanneum Graz, Graz, Austria; ^5^Department of Radiology, Medical University of Graz, Graz, Austria; ^6^Department of Neurosurgery, Medical University of Graz, Graz, Austria

**Keywords:** number processing, arithmetic, parietal cortex, rTMS, intraparietal sulcus, angular gyrus, strategy

## Abstract

The triple-code model (TCM) of number processing suggests the involvement of distinct parietal cortex areas in arithmetic operations: the bilateral horizontal segment of the intraparietal sulcus (hIPS) for arithmetic operations that require the manipulation of numerical quantities (e.g., subtraction) and the left angular gyrus (AG) for arithmetic operations that require the retrieval of answers from long-term memory (e.g., multiplication). Although neuropsychological, neuroimaging, and brain stimulation studies suggest the dissociation of these operations into distinct parietal cortex areas, the role of strategy (online calculation vs. retrieval) is not yet fully established. In the present study, we further explored the causal involvement of the left AG for multiplication and left hIPS for subtraction using a neuronavigated repetitive transcranial magnetic stimulation (rTMS) paradigm. Stimulation sites were determined based on an fMRI experiment using the same tasks. To account for the effect of strategy, participants were asked whether they used retrieval or calculation for each individual problem. We predicted that the stimulation of the left AG would selectively disrupt the retrieval of the solution to multiplication problems. On the other hand, stimulation of the left hIPS should selectively disrupt subtraction. Our results revealed that left AG stimulation was detrimental to the retrieval and online calculation of solutions for multiplication problems, as well as, the retrieval (but not online calculation) of the solutions to subtraction problems. In contrast, left hIPS stimulation had no detrimental effect on both operations regardless of strategy.

## Introduction

The ability to attend to numbers is innate to some degree in human beings. Discrimination of small numerosities begins during the first weeks of life (Antell and Keating, 1983). By about 5 months after birth, children already attend to the addition or subtraction of one or two items (Wynn, 1992). As, we become acquainted with exact arithmetic during school, our strategies in dealing with different arithmetic problems differ. Direct retrieval of solutions from long-term memory is efficient when solving simple addition and multiplication problems that were taught by rote learning. On the other hand, procedural strategies such as counting (“online calculation”) are often used for subtraction, which is often taught by quantity-based counting or other strategies (e.g., inverse addition; Siegler, 1988; Dehaene et al., 2003). Strategy selection, however, depends on several problem-related variables, such as problem size, and individual-related variables, such as working memory span (Imbo and Vandierendonck, 2008). Often, easier problems are solved using retrieval whereas more difficult problems are solved by counting (Zbrodoff and Logan, 2005). A high working-memory span has been linked to the frequent use of retrieval strategies (Imbo and Vandierendonck, 2008).

The triple-code model (TCM) assumes that three different parietal regions are involved in number processing (Dehaene et al., 2003). Based on the findings from neuropsychological, neuroimaging, and brain stimulation studies, the model proposes three distinct and task-specific brain areas in the parietal lobe. The bilateral intraparietal sulcus (IPS) is associated with the core quantity system, the left angular gyrus (AG) is believed to be involved in the verbal processing of numbers, and the posterior superior parietal area in spatial and non-spatial attention (Dehaene and Cohen, 1997; Dehaene et al., 2003). In healthy individuals, arithmetic operations that require online numerical processing such as in simple subtraction and complex (double-digit) addition or multiplication elicited significant unilateral or bilateral IPS activation particularly in its horizontal segment (hIPS; Chochon et al., 1999; Lee, 2000; Menon et al., 2000; Zago et al., 2001; Simon et al., 2002; Delazer et al., 2003, 2005; Ischebeck et al., 2006; Prado et al., 2011; Klein et al., 2013b; De Visscher et al., 2015). The results from these imaging studies seem to support the proposal of the TCM that the hIPS subserve the mental manipulation of numerical quantities (Klein et al., 2013b). This hypothesis was further supported by neuropsychological data showing that pathological lesions of the left and right hIPS caused specific deficits in subtraction with preserved knowledge of rote-learned arithmetic facts (Dehaene and Cohen, 1997; Cohen et al., 2000). Furthermore, findings from non-invasive brain stimulation studies also added evidence that highlighted the importance of the hIPS for arithmetic operations that require online calculation. For example, a virtual lesion-induced on either the right or left hIPS using high frequency repetitive transcranial magnetic stimulation (rTMS) temporarily impaired the participants’ ability to solve double-digit addition and subtraction (Göbel et al., 2006; Andres et al., 2011; Montefinese et al., 2017). Cathodal transcranial direct current stimulation (tDCS) over the left posterior parietal cortex also decreased the learning rates for subtraction, whereas anodal tDCS showed an improvement that lasted over 24 h after stimulation (Hauser et al., 2013; Grabner et al., 2015).

Concerning multiplication, the TCM proposes the involvement of the left AG in the retrieval of arithmetic facts which are represented verbally in long-term memory (Dehaene et al., 2003; Klein et al., 2013b). Indeed, significant left AG activation has been reported when healthy individuals encounter low-interfering problems (e.g., simple addition or single-digit multiplication) that are strongly encoded in long-term memory (Stanescu-Cosson et al., 2000; Grabner et al., 2009; Jost et al., 2009; Klein et al., 2013b; De Visscher et al., 2015; Soylu and Newman, 2016). Incorrect or “confusion” equations in which the proposed answer was true for the other operation (e.g., 9 × 6 = 15) also elicited increased activation in the left AG because the confusion effect automatically (automatic mapping of the operands of the problems and the associated solutions) activates arithmetic facts in memory (Grabner et al., 2013). Multiplication training also led to increased activation in the left AG due to the shift from quantity-based processing to more automatic retrieval (Ischebeck et al., 2006). Moreover, brain lesions located close to the left AG were shown to induce acalculia for addition, multiplication, and division but with spared subtraction (Lampl et al., 1994; Dehaene and Cohen, 1997; Cohen et al., 2000; Lee, 2000). The findings from invasive and non-invasive brain stimulation studies also support a role of left AG in multiplication. Single-session of anodal tDCS over the right AG elicited bilateral AG activity detected with fMRI for multiplication problems rehearsed during stimulation (Clemens et al., 2013). On the other hand, calculation mapping with 5 Hz rTMS was able to induce a maximum error rate (ER) of 30% in the left AG for a single-digit multiplication task (Maurer et al., 2016). Similarly, direct cortical stimulation (DCS) close to the left AG in patients with tumors in the left parietal area disrupted the performance in single-digit addition, subtraction, and multiplication (Whalen et al., 1997; Duffau et al., 2002; Kurimoto et al., 2006). In a patient with a low-grade glioma in the right temporal cortex, DCS of the right AG also disrupted single-digit subtraction (Yu et al., 2011). Moreover, in some cases, removal of the tumor improved multiplication ability (Kurimoto et al., 2006).

Taken together, the mentioned studies support the direct involvement of the AG in arithmetic operations that need retrieval from memory like multiplication and of the hIPS in arithmetic operations that require online calculation like subtraction. However, findings that challenge this anatomical and functional dissociation of these operations also exist. For instance, a PET study failed to show significant activations on either the left and right AG in the retrieval vs. compute contrast (Zago et al., 2001). Several fMRI studies also showed that retrieval and calculation are not exclusive functions of the left AG and hIPS and a reversal or overlap of function may occur (Fulbright et al., 2000; Delazer et al., 2003; Andres et al., 2011; Arsalidou and Taylor, 2011; Rosenberg-Lee et al., 2011; De Visscher et al., 2015). Common activation patterns distributed in frontoparietal and central regions were also reported when contrasting all arithmetic operations of different complexity. It was suggested that this common activation pattern reflects a basic anatomical substrate of working memory, numerical knowledge, and processing based on finger counting that is derived from a network originally related to finger movements (Fehr et al., 2007). Moreover, findings from lesion and brain stimulation studies added controversial results. Intraoperative DCS during complex 2-digit integer minus 1-digit integer subtraction and single-digit multiplication in both the left AG and left hIPS yielded a similar disruption of processing for both operations in four tumor patients (Pu et al., 2011). Preserved multiplication ability was also reported in a patient with damage to the left AG (van Harskamp et al., 2002). In TMS studies, although low frequency (5 Hz) stimulation of the left and right AG induced 30% and 40% errors in simple multiplication and subtraction, respectively (Maurer et al., 2016), high frequency (10 Hz) rTMS also significantly impaired the performance in complex addition when delivered to the left AG (Göbel et al., 2006). In another study, single-pulse TMS stimulation of the bilateral hIPS disrupted the performance in single-digit addition, while only left hIPS stimulation disrupted single-digit multiplication (Salillas et al., 2012). For tDCS, although bilateral bi-cephalic stimulation of the IPS affects magnitude processing, it does not affect double-digit addition and subtraction task performance (Hauser et al., 2013; Klein et al., 2013a). Moreover, single-session anodal tDCS of the left AG enhanced the RT and decrease the solution rates for large and small addition and subtraction problems, respectively (Rütsche et al., 2015). This overview demonstrates that the complete anatomical and functional dissociation of arithmetic operations in the parietal cortex is far from being clear.

One of the possible reasons for this contradictory pattern of results is the disregard for different strategy use in solving arithmetic problems. Item-by-item strategy use was not fully and correctly accounted for by previous studies. Instead, the two operations, subtraction, and multiplication were commonly used to tap into the brain networks subserving the mental manipulation of numerical quantities and arithmetic fact retrieval, respectively. However, this simple distinction might not be valid for all items. For example, ties (e.g., 3 × 3, 3 + 3) are often solved faster than other problems, which has been attributed to direct memory retrieval (Imbo et al., 2007). It has also been assumed that, in the case of single-digit addition problems, retrieval of arithmetic fact knowledge is used only for rather small problems (e.g., 2 + 3) but not for relatively larger problems (e.g., 8 + 9; Stanescu-Cosson et al., 2000; Klein et al., 2013b). Additionally, retrieval might again be the strategy of choice for multi-digit problems such as 12 + 12 or 20 + 30. This also applies to single-digit multiplication problems because multiplication with zero and small problems are assumed to be solved by rule application and fact retrieval, respectively, and problems with large operands sometimes involve backup strategies when direct retrieval is not sufficient (Jost et al., 2009). Therefore, the majority of the previous studies underestimated the impact of strategy use on an item-by-item basis. Averaging of response latencies across trials that involved different strategies might result in misleading conclusions about how adults solve arithmetic problems (Thevenot et al., 2007). The same critique applies to recent neuroimaging studies. Currently, only one fMRI study (Grabner et al., 2009) has utilized trial-by-trial self-reports to assess strategy usage. So far, no noninvasive brain stimulation study has used this approach to systematically explore the impact of strategy use in subtraction and multiplication. Elucidating the anatomical and functional dissociation of subtraction and multiplication to distinct areas of the parietal cortex will extend our knowledge about the neuronal circuits involved in arithmetic operations. This is useful in understanding the course of disorders like developmental dyscalculia which affects 5–6% of school children, as well as, in formulating interventions for an acquired numerical disability such as in the elderly (Shalev, 2004; Nouchi and Kawashima, 2014).

The present study addressed this issue by using an item-by-item questionnaire to investigate the extent to which the participant’s strategy usage affects the anatomical dissociation of multiplication and subtraction. First, we used fMRI to identify the parietal cortex areas recruited during the performance of subtraction and multiplication for each participant. Second, the participants underwent rTMS sessions during which an inhibitory stimulation paradigm (1-Hz rTMS) was applied over three target areas: the left hIPS, left AG, and the vertex as a control site. Participants solved subtraction and multiplication problems before, during, and after stimulation. Immediately after each experimental session, participants were asked to indicate which strategy (online calculation or retrieval) they used to solve each problem using a questionnaire. We predicted that if the left hIPS is engaged in subtraction, the rTMS-induced virtual lesion would increase the solution latency of trials solved by online calculations. On the other hand, if the left AG is engaged in multiplication, the rTMS-induced virtual lesion will increase the solution latency of trials solved by retrieval.

## Materials and Methods

### Participants

The number of participants was determined *a priori* using the statistical software G*Power 3.1.9 (Faul et al., 2007). The estimation indicated that 12 participants would be sufficient in a within-subject repeated measure design (power level of 95% and medium (0.50) effect size). In the study, 16 healthy young volunteers (seven males) with a mean age of 26.25 ± 7.07 (SD) years were recruited. They all had a normal or corrected-to-normal vision and were right-handed according to the Edinburgh Handedness Inventory (Oldfield, 1971). Participants neither had a history of acute or chronic medical or neuropsychiatric diseases and contraindications to TMS such as metallic or electrical implants in the body (Rossi et al., 2009). They received monetary compensation for their participation and gave written informed consent before the experiment. The study protocols complied with the guidelines of the Declaration of Helsinki for human studies and were approved by the ethics committee of the Medical University Graz.

### Stimuli and Task

**Figure 1:**
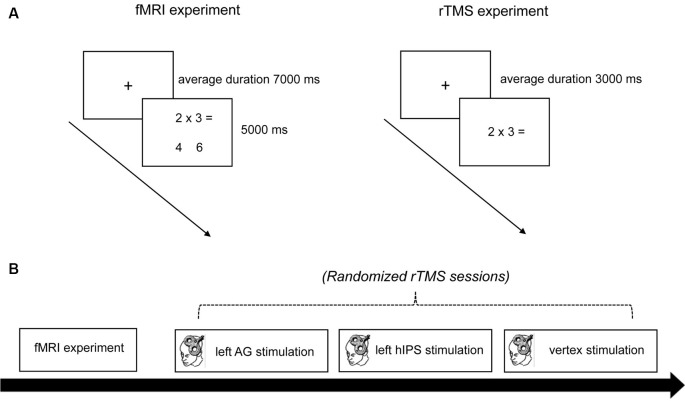
Experimental procedure. **(A)** Trial timing schema of the fMRI and rTMS experiment. In the fMRI experiment, a trial started with the presentation of a fixation cross for an average duration of 7,000 ms. Then the problems (with the solution and distractor) appeared for 5,000 ms. In the rTMS experiments, a trial started with the presentation of a fixation cross for an average duration of 3,000 ms. Then the problems (without the solution and distractor) appeared until the triggering of the voice-key. **(B)** Time course of the experiment. First, anatomical and functional MRI data sets were obtained. Then the participants underwent three sessions of rTMS stimulation (left AG, left hIPS, and vertex) in randomized order. rTMS was applied at 1 Hz for 15 min (900 pulses). fMRI, functional magnetic resonance imaging; rTMS, repetitive transcranial magnetic stimulation; AG, angular gyrus; hIPS, horizontal segment of the intraparietal sulcus.

In the fMRI and rTMS experiments, we presented 36 subtraction and 36 multiplication problems. The problems were presented horizontally in white on a black background using Presentation software (Neurobehavioral Systems Inc., Berkely, CA, USA) for the fMRI experiment and Superlab 4.5 software (Cedrus Corporation, San Pedro, CA, USA) for the rTMS experiment (Figure 1A). For multiplication problems, one-digit × one-digit multiplications with the numerals from 2 to 9 were selected, including ties. Problems with two different numerals (e.g., 2 × 3) were always presented with the smaller number as the first operator. For subtraction problems, one-digit numerals were subtracted from tens, always requiring a carry operation (e.g., 15–8, but not 15–3).

For the fMRI experiment, the problems were randomized and presented once in a single block (72 trials). The problems were presented together with the solution and a distractor. For multiplication, the distractor was the result of an operant-related multiplication problem. For subtraction, the distractor was either one or two units away from the solution. The distractor was presented on the left side for half of the problems, and on the right side for the other half. After the presentation of the problems and two result alternatives, participants had to press the left or right button to indicate which of the two presented numbers was the solution. During the rTMS experiment, tasks and stimuli were the same as in the fMRI experiment. However, the problems were presented without result alternatives. The participants were asked to mentally solve the problems and speak the solution into a head-mounted microphone connected to a voice-key device. For each rTMS session, the participants solved the 72 problems five times [once before, during, and after (0 min, 30 min, and 60 min) stimulation]. Therefore, one rTMS session had a total number of 360 trials.

### Functional Magnetic Resonance Imaging (fMRI)

MRI images were acquired on a 3.0 Tesla whole-body system Siemens Skyra scanner with an echo-planar capable gradient system together with a 20-channel birdcage head coil (Siemens Medical Systems, Erlangen, Germany). For each participant, an anatomical 3-D scan based on a T1-weighted sequence was recorded (TR/TE = 1,650 ms/1.82 ms, matrix = 256 × 256, FOV = 256 mm, 192 sagittal slices, in-plane resolution: 1 mm × 1 mm, slice thickness: 1 mm, 0.5 mm gap). The anatomical scan was followed by functional measurements. For the functional images, a T2*-weighted echo-planar imaging (EPI) sequence was used (TR = 2,000 ms TE = 25 ms, matrix = 74 × 74, FOV = 224 mm, 38 axial slices, in-plane resolution: 3 mm × 3 mm, slice thickness: 3 mm, 0 mm gap) which is sensitive to blood-oxygen-level-dependent (BOLD) contrasts.

### Repetitive TMS (rTMS)

The stimulation was performed using a MagPro X100 stimulator with MagOption (MagVenture GmbH, Denmark). Single and repetitive biphasic TMS pulses were delivered using the MCF-B65 and C-B60 figure-of-eight MagVenture coils, respectively. Both coils have a 75 mm diameter on one winding. For stable and precise positioning of the magnetic coil above the areas of interest, the Localite TMS Navigator (Localite GmbH, Sankt Augustin, Germany) system tracks the sensors attached to the coil concerning the adhesive reflectors on the patient’s forehead using an infrared tracking device (Polaris Spectra, Northern Digital Inc., Waterloo, ON, Canada). The stimulation intensity was set at 110% of the individual participant’s active motor threshold (AMT) determined from the primary motor cortex representation of the right abductor pollicis brevis (APB) muscle using single-pulse TMS. Electromyography (EMG) recordings from the right APB muscle were obtained using surface electrodes with a belly-tendon montage. AMT was defined as the minimum stimulus intensity that elicits a motor-evoked potential (MEP) response of >100 μV (peak-to-peak) during moderate spontaneous background muscle activity (~10% of the maximum voluntary contraction) in at least five of ten consecutive trials (Rossini et al., 1999). During rTMS stimulation, magnetic pulses were delivered at a frequency of 1 Hz for 15 min (900 pulses; Houdayer et al., 2008). The magnetic coil was held perpendicular to the left hIPS and left AG and was oriented on the central plane at the vertex. All stimulation parameters conformed to the safety guidelines for rTMS (Wassermann, 1998; Rossi et al., 2009).

### Experimental Procedure

The study was conducted in a single-blinded, randomized design with an active TMS control condition. Each participant underwent one fMRI and three randomized rTMS sessions (Figure 1B). The study always began with the fMRI session. During fMRI, the participants lay supine in the scanner and their head was stabilized with foam paddings. They wore earplugs to protect them from the scanner noise. A computer outside the scanner room controlled the stimulus presentation and scanner triggering (Neurobehavioral Systems Inc., Berkely, CA, USA). The participants viewed the stimulus projected from a monitor at the head end of the scanner on a mirror mounted on top of the head coil. In the fMRI session, each trial started with the presentation of a fixation cross for a jittered duration of 3–11 s (in 500 ms steps, average duration 7 s). Subsequently, the problems with the solutions and distractors appeared for 5 s (Figure 1A). Reaction times (RTs) were measured from the onset of the problem presentation until a button press. All 72 trials were presented (without pause) in a single block, leading to a total duration of approximately 13 min. A minimum of 5 days separated the fMRI and the first rTMS session.

All participants underwent three sessions of rTMS stimulation separated by an interval of at least 7 days to avoid carry-over effects. The stimulations were performed in all participants in the middle of the day between 1:00 and 5:00 pm. The stimulation targets (left AG, left hIPS, and vertex) were randomized for each participant. They were not informed about the target locations for each experimental session and the neuronavigation monitor was placed out of their sight to ensure efficient blinding. Vertex stimulation served as the control condition since previous rTMS studies showed that stimulation of this site did not affect number processing (Dormal et al., 2008, 2012; Andres et al., 2011). Additionally, vertex stimulation reproduces the somatosensory effects of parietal stimulations and is considered a better control than other sham stimulation alternatives (Robertson et al., 2003; Dormal et al., 2012). Furthermore, to control for unspecific effects of the stimulation (e.g., motor area), participants performed a grooved pegboard test (PBT) before the first rTMS experimental session and immediately after the last arithmetic task performance (60 min after stimulation) in the third rTMS experimental session (Koch and Rothwell, 2009; Koch et al., 2009; Feurra et al., 2011; Rivera-Urbina et al., 2015).

During rTMS sessions, participants were seated in a comfortable chair with head and armrests. They were informed about the sensations during TMS stimulations and were assured that any calculation impairment would be temporary. The experiment started once all questions were answered. First, we performed the coregistration of the participant’s head and the participant’s 3D T1-weighted MRI scan. The high-resolution T1 MRI data were loaded into the Localite TMS Navigator System. For the tracking device to locate the individual head and the position of the TMS coil during stimulation, three reflective sphere markers were attached to the patient’s forehead and TMS coil. Subsequently, three anatomical landmarks (the nasion, left, and right outer canthus) were marked in the 3D MRI image. Using a digitizing pen that also contained sphere markers, the same anatomic landmarks were marked on the patient’s real head. To further improve the co-registration quality, an additional 200 anatomical landmarks were added on the patient’s head by tracing the scalp with the digitizing pen. To ensure the goodness of fit (patient’s real head and structural MRI), we kept the root mean squared error of the fitting procedure at less than 2.5 mm for all participants. The co-registration created a 3D head model in which the peeling depth could be individually adjusted to visualize the cortical surface.

After the coregistration, the “motor hotspot” or the primary motor cortex representation of the right APB muscle was located using anatomical landmarks (e.g., hand knob at the precentral gyrus). The “motor hotspot” was defined as the cortical location where the lowest stimulator output elicited the biggest MEP amplitudes. EMG electrodes were attached at the right APB muscle in a belly-tendon montage to monitor the MEP amplitudes during stimulation using the built-in EMG device in the stimulator. The participants wore earplugs to shield them from the noise of the stimulator. To confirm the location of the motor hotspot, single-pulse TMS stimulation was applied at a frequency of 0.25 Hz while monitoring MEP amplitudes. The coil was placed tangentially to the scalp at an angle of 45° to the midsagittal plane with the handle pointing laterally and posteriorly generating an anteroposterior current direction in the brain. The participants were asked to briefly and voluntarily contract the APB muscle (~10% of the maximum voluntary contraction) while TMS was delivered. The stimulation intensity was gradually reduced until the AMT was reached. Participants with an individual AMT beyond 50% of the maximum stimulator output would have been excluded from the experiment (none). Subsequently, the participant’s functional data set was overlaid on the 3D reconstruction. Cortical areas with significant BOLD activations [“fMRI hotspots” or regions of interest (ROI)] were identified and marked.

For the rTMS experiment, each trial started with the presentation of a fixation cross for a variable duration between 2,000 ms and 4,000 ms (in steps of 250 ms, average duration: 3,000 ms). This was followed by the presentation of one of the 72 arithmetic problems (36 subtraction, 36 multiplication). The participants were asked to mentally solve the problem and speak the solution aloud into a head-mounted microphone connected to a voice-key. The problem disappeared on the triggering of the voice-key. After voice-key triggering, the participant’s answer was recorded by the experimenter, or a code (“0”) for voice-key failure was recorded. The participants solved the arithmetic problems once before, during, and after (0 min, 30 min, and 60 min) stimulation. RT was measured from the onset of the problem presentation until the triggering of the voice key. After each session, participants completed a questionnaire of the 72 arithmetic problems. For each arithmetic problem, they were instructed to tick a box to indicate whether they retrieved the answer from memory or whether they had to calculate. Including the preparation time (20 min), each rTMS experimental session lasted for about 120 min.

## Data Analysis

### fMRI Data

Data pre-processing and analysis were performed with SPM12 (Wellcome Department of Cognitive Neurology, London, UK). The first two functional scans of each participant were discarded to allow for signal stabilization. The functional scans were motion-corrected and unwrapped. They were normalized using the MNI functional (EPI) template. Finally, images were spatially smoothed with a Gaussian kernel of 8 mm FWHM. Statistical analyses were performed based on the general linear model as implemented in SPM12. First, a model with two conditions (subtraction/multiplication) was analyzed. To investigate the influence of strategy (calculation or retrieval) participants were asked to complete a questionnaire including all problems before the first rTMS session. For the fMRI analysis, these data were then used to estimate a second model with two conditions (calculation/retrieval). The canonical form of the hemodynamic response function and its first temporal derivative was used for modeling. The motion parameters gained from the motion correction procedure were entered into the model as parameters of no interest. A high-pass filter (cut-off frequency: 1/120 Hz) was applied to remove low-frequency drifts. No global normalization was used. A second level or random-effects analysis was calculated based on the contrast images of the individual subjects (Friston, 1999). The statistical parameter maps were thresholded using an initial uncorrected *p*-value threshold of less than 0.001, reporting only clusters as significant when they had a corrected *p*-value of less than 0.05 on the cluster level. The correction of the *p*-level was based on continuous random field theory as implemented in SPM12 [family-wise error (FWE) corrected].

### Behavioral Data (Questionnaire)

Participants had selected either retrieval or calculation as their strategy in the questionnaire, which contained all 72 problems and was administered once after every rTMS session. Only correctly ticked problems were analyzed (3,452 out of 3,456 data points). The percentages for the retrieval strategy were entered into a repeated-measures ANOVA with the operation of the within-subjects factors (subtraction, multiplication) and session (one, two, or three).

### Behavioral Data (Reaction Time and Error Rate)

Statistical analyses were conducted separately for the raw RTs and error rates (ERs) during fMRI and rTMS sessions using SPSS software (SPSS 24, IBM Corp., Armonk, NY., USA). In the final analysis, only the RTs from correctly answered and ticked problems were included. Trials for which the RTs were outside of +2 standard deviations and trials with RTs below 300 ms or longer than 5,000 ms (outliers) were excluded. Grouping the RTs according to strategy type produced unbalanced data sets. Therefore, we decided to analyze the RT from the fMRI and three rTMS sessions using a linear mixed-effects model (LMM) because this analysis can accommodate data sets with different numbers of observations per subject (West, 2009). In the models, each participant was specified as a random factor (random intercept model). The RT or ER served as the dependent variable. For the fMRI data sets, a full model included the within-subject factor “operation” (multiplication and subtraction), and “strategy” (calculation and retrieval) as fixed factors. On the other hand, a full model for the rTMS data sets included the within-subject factor “stimulation site” (hIPS, AG, and vertex), “operation” (multiplication and subtraction), “strategy” (calculation and retrieval), and “time” (before, during, and 0, 30 and 60 min after stimulation) as fixed factors.

Normal data distribution (Shapiro–Wilk test) and homogeneity of variance tests (Levene’s test) were conducted. To achieve a parsimonious model for the data, we conducted a (forward) stepwise approach by incrementally adding the predictors to a baseline model (Barr et al., 2013). The baseline models only contained the random factor (intercept) to examine the individual variation in the dependent variable without regard to the other predictors (Singer and Willett, 2003). We then added the within-subject factors including their respective interactions to the model one at a time and compared the Akaike Information Criterion (AIC) values that indicate model adequacy. Model over-fitting, particularly for RTs from the rTMS experiment, can be minimized using this method because it penalizes the likelihood function for having too many parameters. Upon the addition of a factor, a decrease or increase in AIC value (>2) indicates model fit improvement or worsening, respectively (Burnham and Anderson, 2002). A maximum likelihood estimation (Compound Symmetry models) was used to estimate the parameters of each model. Additionally, we determined the Akaike weight of each model because the AIC value only compares one model to the next and does not indicate the absolute fit of the model to the data (Burnham and Anderson, 2002). The Akaike weights compare all possible models and determine which model fits the data best for all comparisons. In the final models, we also excluded non-significant factors except when they were involved in significantly higher interactions. Additionally, to test for multicollinearity, we also determined the tolerance and variance inflation factor of the final models. SPSS does not provide effect size values for mixed models, we therefore manually calculated Cohen’s d as a measure of effect size. Significant findings from the models were explored using paired *t*-tests for *post hoc* comparisons (two-tailed, *p* < 0.05, Bonferroni corrected for multiple comparisons). A *t*-test for dependent measures was used to compare the grooved PBT performance before and after the experiment. A *p*-value of < 0.05 was considered significant for all statistical analyses. All values are expressed as mean ± standard error of the mean (SEM).

## Results

### fMRI Data

**Figure 2:**
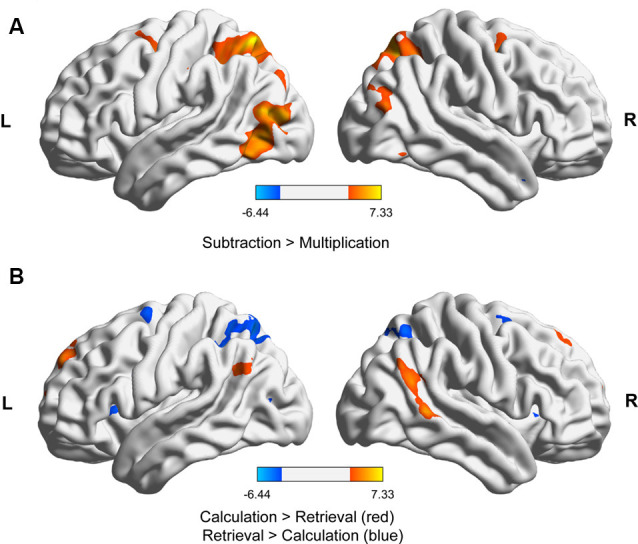
fMRI results for **(A)** subtraction vs. multiplication (both strategies) and **(B)** calculation vs. retrieval (both operations). The activations in the images were thresholded at *p* < 0.001 uncorrected, showing only clusters significant at *p* < 0.05, family-wise error (FWE)-corrected at the cluster level. The images were generated with BrainNet Viewer (Xia et al., 2013).

For the fMRI data, we first contrasted subtraction with multiplication. We found significantly stronger activation for subtraction than for multiplication in the right and left superior parietal lobule, including the IPS and extrastriate visual areas, as well as the right middle frontal gyrus (Figure 2A, Table 1). No brain area was significantly activated in the reverse contrast. To investigate whether brain activation depended on the strategy used we then contrasted problems whose solutions were reported calculated with problems that were retrieved (Figure 2B, Table 1). We again found activations in the right and left superior parietal lobule including the IPS, the left precentral gyrus, and left superior frontal gyrus, as well as in the middle cingulate cortex. In the reverse contrast, there was significant activation in the left AG, the right middle temporal gyrus including the AG, the middle cingulate cortex, as well as in the right and left superior frontal gyrus.

**Table 1:** Brain areas activated for subtraction vs. multiplication (both strategies) and retrieval vs. calculation targets (both operations).

Hemisphere		*x*	*y*	*z*	*K*	*Z*
	**Subtraction > Multiplication**				
Left	Superior parietal lobule	−26	−66	60	4,519	4.95
Right	Superior parietal lobule	16	−72	62	1,785	4.45
Right	Middle frontal gyrus	24	−2	48	1,082	4.28
	**Multiplication > Subtraction**				
	ns.					
	**Retrieval > Calculation**				
Right	Middle cingulate cortex	6	−40	36	778	4.71
Right	Middle temporal gyrus	62	−46	4	598	4.41
Left	Superior frontal gyrus	−14	56	38	225	4.05
Right	Superior frontal gyrus	16	44	52	160	3.95
Left	Angular gyrus	−50	−60	32	141	3.60
	**Calculation > Retrieval**					
Right	Middle cingulate cortex	10	22	40	710	4.39
Left	Superior parietal lobule	−16	−64	60	1,441	4.26
Right	Superior parietal lobule	26	−62	54	310	3.81
Left	Precentral gyrus	−50	6	26	191	3.81
Left	Superior frontal gyrus	−28	0	70	170	3.75

*Statistical parameter maps were thresholded with an initial threshold of *p* < 0.001 uncorrected, reporting only clusters that survived an family-wise error (FWE)-corrected *p*-value < 0.05. Coordinates are reported as given by SPM12 (MNI space). k = cluster size, Z = Z value for the maximally activated voxel of the cluster*.

### Behavioral Data (RT and ER) During fMRI

Participants had calculated simple multiplication and subtraction problems during fMRI measurement. Their answers were categorized according to the strategy used for a solution, as given by the questionnaire administered in the first session of the rTMS experiment. For the RT analysis, 1,090 out of 1,152 problems were correctly answered and considered. Additional 16 trials were considered outliers and excluded from the analysis. One additional data point was lost due to missing questionnaire data. Therefore, the final model for the RT contained 1,073 trials or 93.14% of the whole data set. The analysis of the RT revealed that participants were faster with multiplication problems (1.91 s, SD = 0.75 s) than with subtraction problems (2.36 s, SD = 0.85 s), which led to a significant main effect of operation (*F*_(1,556.74)_ = 38.94, *p* ≤ 0.001, *d* = 0.544). They were also faster for problems when the solution could be retrieved (1.98 s, SD = 0.39 s) than when the solution had to be calculated (2.26 s, SD = 0.39 s), which is reflected in a significant main effect of strategy (*F*_(1,556.07)_ = 15.10, *p* ≤ 0.001, *d* = 0.717). The interaction was not significant (*F*_(1,551.95)_ = 0.007, *p* = 0.935, *d* = 0.108). In the analysis of the ER, no significant effects obtained.

### Strategy Questionnaire in the rTMS Experiment

As skilled adults rely on the multiplication tables, we had hypothesized that retrieval was the predominant strategy in multiplication and less so in subtraction. Furthermore, it was expected that participants more often relied on retrieval the more familiar they got with the problems from the first to the third session due to learning. Both expectations were confirmed by our results. Overall, retrieval was more often used for multiplication (70.14%, SD = 11.37%) than for subtraction (27.20%, SD = 11.81%) yielding a main effect of operation (*F*_(1,15)_ = 44.31, *p* < 0.001, $\eta_{\text{p}}^{2}$ = 0.75). Furthermore, participants used the retrieval strategy more in the later sessions (session 1: 42.97%, SD = 7.91%, session 2: 50.04%, SD = 8.87%, and session 3: 52.60%, SD = 9.11%, main effect session (*F*_(2,30)_ = 5.70, *p* = 0.008, $\eta_{\text{p}}^{2}$ = 0.28). The interaction was not significant. This indicates that familiarity had a similar effect on strategy use for both operations.

### TMS Parameters and Impact on Motor Function

During the TMS sessions, all participants tolerated the single and repetitive TMS stimulations well. The mean stimulation intensities (hIPS: 42.75 ± 1.65% MSO, AG 40.93 ± 1.59% MSO, vertex: 43.12 ± 1.83% MSO) did not significantly differ between the sessions. There were no reports of headaches, dizziness, or nausea. In four participants, we noticed some episodes of difficulty verbally naming the solution for multiplication problems during AG stimulation but they were able to finish the experiments. The results of the grooved PBT indicated that our stimulation protocol had no significant impact on motor function (remote effect) of the right (before: 58.25 ± 1.65 s, after: 57.63 ± 1.64 s, *t*_(15)_ = 0.379, *p* = 0.710) and left hand (before: 60.31 ± 1.88 s, after: 62.69 ± 2.13 s, *t*_(15)_ = −1.488, *p* = 0.158). PBT performance were also comparable between the participants who received vertex (*n* = 6; right hand: 57.66 ± 3.67 s, left hand: 63.16 + 4.96 s), left hIPS (*n* = 5; right hand: 58.20 + 2.72 s, left hand: 63.00 + 2.50 s), and left AG (*n* = 5; right hand: 57.00 + 2.16 s, left hand: 61.80 + 3.30 s) stimulation on their last experimental session (all *p* ≥ 0.05).

### Behavioral Data (RT and ER) in the rTMS Experiment

**Figure 3:**
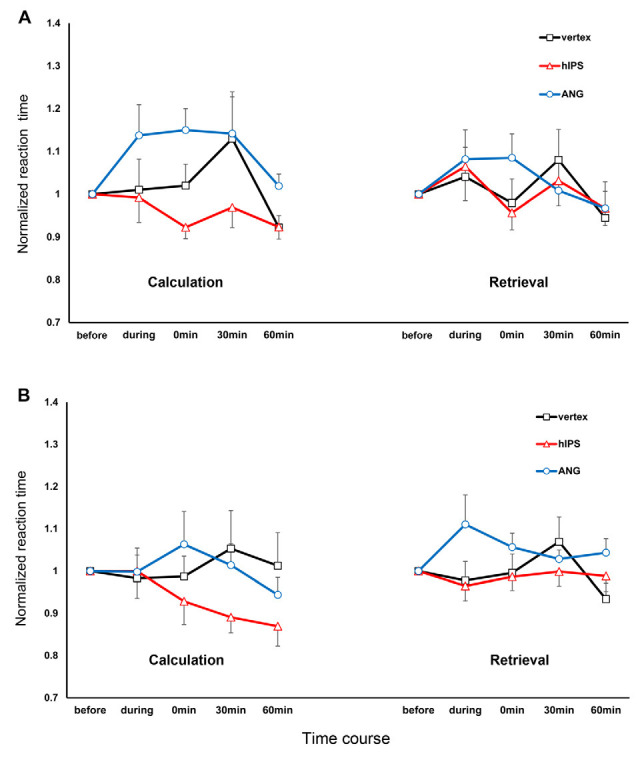
Response times for multiplication and subtraction problems, depending on time and site of stimulation, separated for operation and strategy. The *x*-axis displays the time points. The *y*-axis represents the reaction times (RTs) normalized concerning the respective RT before the stimulation (RT during and after stimulation/RT before stimulation). **(A)** Multiplication: The stimulation of the AG inhibited the online calculation and retrieval of solutions for multiplication problems. The stimulation of the hIPS and vertex did not inhibit online calculation and retrieval. **(B)** Subtraction: stimulation of the AG inhibited the retrieval of solutions to subtraction problems during stimulation. Stimulation of the hIPS had no detrimental effect on online calculation or retrieval. Error bars represent the standard error of the mean. hIPS, the horizontal segment of the left intraparietal sulcus; AG, angular gyrus.

For the RT, we decided to interpret a full model because all the main effects were highly significant (Table 2), the addition of each variable and their interactions improved the model based on the AIC values, and a model containing the 4-way interactions did come out best 100% of the time based on the Akaike weights (Supplementary Table S1). For the three rTMS sessions, we included 93.32% (16,127 trials out of 17,280) of the RT data in the final analysis. The raw data entered in the final model were normally distributed after log transformation (Shapiro–Wilk test) and the variances were equal (Levene’s test; all *p* > 0.05). Multicollinearity was not a concern in the final model since the tolerance range and variance inflation factors were 0.863–1.00 and 1.000–1.159, respectively. The RT data from the three rTMS sessions are presented in Figures 3A,B. These data are normalized to their respective baseline measures to remove baseline differences between the sessions. The results of the analysis (performed on the raw data, not normalized to baseline data) revealed significant differences in RT before and after rTMS stimulation of the three target areas (significant main effect of time: *F*_(4,16111.02)_ = 5.07, *p* ≤ 0.001, *d* = 0.078; significant main effect of stimulation site: *F*_(2,16111.27)_ = 23.11, *p* ≤ 0.001, *d* = 0.175; and significant time and stimulation site interactions: *F*_(8,16111.25)_ = 4.40, *p* ≤ 0.001, *d* = 0.214; Figures 3A,B). The *post hoc* comparisons for the factor time showed that participants were significantly faster in solving arithmetic problems 60 min after stimulation compared to before (*p* = 0.001) and during (*p* = 0.030) stimulation. They were specifically faster in solving arithmetic problems when the left hIPS was stimulated compared to the vertex (*p* ≤ 0.001) and AG (*p* ≤ 0.001; pairwise comparisons, Bonferroni corrected; Figures 3A,B). The analysis also showed that the participants were slower in solving multiplication than subtraction problems (significant main effect of operation: *F*_(1,16116.85)_ = 112.73, *p* ≤ 0.001, *d* = 0.350) and slower in retrieving the answer compared to calculating it particularly 60 min after stimulation (significant main effect of strategy: *F*_(1,16126.54)_ = 434.45, *p* ≤ 0.001, *d* = 0.775; and significant time and strategy interactions: *F*_(4,16111.01)_ = 5.25, *p* ≤ 0.001, *d* = 0.159). Concerning the site-specific effect, rTMS stimulation of the left AG slowed down the online calculation or retrieval process in both operations (significant operation and strategy interactions: *F*_(1,16122.48)_ = 45.40, *p* ≤ 0.001, *d* = 0.350; significant strategy and stimulation site interactions: *F*_(2,16111.99)_ = 6.26, *p* = 0.002, *d* = 0.185). In contrast, similar to the vertex stimulation, rTMS of the hIPS did not inhibit the online calculation and retrieval of answers to both multiplication and subtraction problems.

**Table 2:** Results of the linear mixed model (LMM) performed on the reaction times from the repetitive transcranial magnetic stimulation (rTMS) experiment.

	Numerator *df*	Denominator *df*	*F*-value	*p-*value	Cohen’s *D*
Time	4	16,111.02	5.07	<0.001*	0.078
Operation	1	16,116.85	112.73	<0.001*	0.350
Strategy	1	16,126.54	434.45	<0.001*	0.775
Stimulation site	2	16,111.27	23.11	<0.001*	0.175
Time × operation	4	16,111.00	0.79	0.534	0.096
Time × strategy	4	16,111.01	5.25	<0.001*	0.159
Time × stimulation site	8	16,111.25	4.40	<0.001*	0.214
Operation × strategy	1	16,122.48	45.40	<0.001*	0.350
Operation × stimulation site	2	16,111.21	1.78	0.164	0.175
Strategy × stimulation site	2	16,111.99	6.26	0.002*	0.185
Time × operation × strategy	4	16,111.00	0.75	0.555	0.229
Time × operation × stimulation site	8	16,111.00	0.22	0.987	0.153
Time × strategy × stimulation site	8	16,111.02	1.71	0.090	0.176
Operation × strategy × stimulation site	2	16,111.82	2.52	0.081	0.189
Time × operation × strategy × stimulation site	8	16,111.00	1.26	0.262	0.184

*For the LMM (random intercept model), each participant was treated as a random factor. The within-subjects factor stimulation site (hIPS, AG, and vertex), operation (multiplication and subtraction), strategy (calculation and retrieval), and time (before, during, and 0, 30 and 60 min after stimulation) were treated as fixed factors. Asterisks indicate significant results (*p* < 0.05). df = Degrees of freedom*.

Regarding the ERs, the participants exhibited very low ERs before the stimulation (4.44% in multiplication and 4.46% in subtraction). The ER further decreased after stimulation in all conditions as indicated by the significant main effect of time only (*F*_(4,358.79)_ = 3.66, *p* = 0.006, *d* = 0.420; Supplementary Table S2).

## Discussion

The present study aimed at elucidating the anatomical and functional dissociation of subtraction and multiplication into distinct parietal cortex areas namely the left hIPS and left AG, respectively. First, we identified brain areas with significant activation during the performance of subtraction and multiplication using fMRI. Second, these brain areas were stimulated using rTMS. We were particularly interested in the impact of the participant’s strategy of choice on the dissociation of these two operations. Therefore, we used a strategy questionnaire to have first-hand knowledge of how the participants solved subtraction and multiplication problems. The strategy questionnaires alone revealed that multiplication compared to subtraction problems were more often solved using a retrieval strategy. fMRI data analysis revealed significant recruitment of the left AG during retrieval (more than online calculation), even though we did not observe a significant increase in activity at the left AG during multiplication (compared to subtraction). Conversely, we observed stronger activation in the bilateral hIPS during subtraction (more than in multiplication) and online calculation (more than for retrieval). Our fMRI findings corroborate the results of previous imaging studies highlighting the role of the left AG in multiplication problems that require retrieval strategy and the bilateral hIPS for subtraction problems that require online calculation strategy (Delazer et al., 2003, 2005; Ischebeck et al., 2006; Grabner et al., 2009). Additionally, our imaging results also showed significant activations of the prefrontal, frontal, and cingulate cortices during calculation and retrieval. Activations of these areas indicate their involvement in the strategy selection network in number processing that requires working memory, strategic organization during encoding, decision making, and response selection (Taillan et al., 2015). In the rTMS sessions, our results showed that left AG stimulation was detrimental to the retrieval and online calculation of solutions for multiplication problems, as well as, the retrieval (but not online calculation) of the solutions to subtraction problems. In contrast, left hIPS stimulation had no detrimental effect on both operations regardless of strategy.

### RTMS Stimulation of the Left AG

The stimulation of the left AG resulted in marked RT slowing in multiplication (more than subtraction) problems which indicate an impairment in our participants’ ability to perform this arithmetic operation. Our result provides further support for the assumption that the left AG is crucial in solving arithmetic problems that are typically solved by the retrieval of the solution from verbal long-term memory (Cohen et al., 2000; Dehaene et al., 2003; Seghier, 2013; Andin et al., 2015). However, when we analyzed the RT based on strategy, the results were contrary to our expectations because the impairment was smaller in magnitude for multiplication problems solved using retrieval compared to the online calculation. Retrieval was only impaired during and immediately after the stimulation, while online calculation was impaired until 30 min after stimulation. Furthermore, we also observed impairment in the retrieval of the solutions to subtraction problems, particularly during the stimulation. Therefore, our results suggest that the left AG plays a role in the retrieval of the solution from memory for both multiplication and subtraction problems. Our results further suggest that the left AG is also responsible for the online calculation of solutions to multiplication problems.

The impairment in retrieving the solution to multiplication problems was expected because retrieval of overlearned multiplication facts (e.g., 2 × 3) is supported by language-relevant areas that include the left AG (Dehaene et al., 2003). This is demonstrated among adult individuals with deficits in phonological processing, such as those with developmental dyslexia who have prominent difficulties in multiplication due to poor retrieval of arithmetic facts (De Smedt and Boets, 2010). This is because arithmetic facts are represented verbally in long-term memory, allowing such problems to be solved by arithmetic fact retrieval (Klein et al., 2013b). In our study, the close functional interplay of arithmetic fact retrieval and language processing was demonstrated in four participants who exhibited difficulties to verbalize the result for multiplication problems during left AG stimulation. The interference probably involved a genuine impairment of arithmetic fact retrieval because the production task (verbal response) put stronger demands on the retrieval of the correct answer from memory than the solution selection task used for the fMRI experiment (Dehaene et al., 1999; Andres et al., 2011). We can rule out the possibility that the speech interruptions were due to motor impairment because the effect was specific to the stimulation of left AG during multiplication. Also, the stimulation had no impact on grooved PBT performance. The impairment in the retrieval of the solution to subtraction problems could be anticipated because some subtraction problems (e.g., 12–6) may be stored in verbal long-term memory as well. Indeed, impairment in single-digit addition, subtraction, and multiplication can be elicited by directly stimulating the cortical areas close to the left AG during tumor surgery (Whalen et al., 1997; Duffau et al., 2002; Kurimoto et al., 2006). In our arithmetic task, even though we did not use single-digit operands, retrieval might have been the strategy of choice for some subtraction problems such as in 16–8 because 16 is double the amount of 8. To conclude, the impairment in the retrieval of solution for both operations indicates that the interference in automatic fact retrieval is due to the rTMS-induced tonic suppression of neuronal activity in the left AG (Ridding and Ziemann, 2010).

One might ask if arithmetic operations solved by retrieval involve the left AG (Dehaene et al., 2003), why is the retrieval not fully disrupted by the stimulation? The AG has strong functional and anatomical connectivity with the hippocampal system and the frontal areas mainly *via* the middle longitudinal fascicle (ventral pathway). This is different from the IPS, which is connected by the superior longitudinal fascicle (dorsal pathway) with frontal areas for magnitude-related processes, as revealed by probabilistic fiber tracking (Klein et al., 2013b). Additionally, dorsal fiber tracts like the cortical cingulate route (via retrosplenial cortex) that provide an indirect pathway for hippocampal interactions with prefrontal cortex were also described to subserve arithmetic fact retrieval (Uddin et al., 2010; Klein et al., 2013b; Bubb et al., 2017). The retrosplenial cortex was reported to be involved in the recognition of familiar objects and procedures, as well as autobiographical memory. This function is related to the retrieval of familiar arithmetic facts from memory (Vann et al., 2009; Sestieri et al., 2010, 2013; Klein et al., 2013b). Possibly the left AG stimulation might not have been sufficient to completely inhibit the retrieval process since other brain areas (e.g., retrosplenial cortex) subserving memory retrieval were less affected by the inhibitory effect of the stimulation. This is because the inhibitory effect of 1-Hz rTMS is mainly localized in the cortex being stimulated which in our case was the left AG. As shown in *in vivo* electrophysiological studies in the human motor cortex, 1-Hz rTMS only suppresses the late I-waves that depend on the excitability of motor cortico-cortical circuits (Di Lazzaro et al., 2003, 2010; Cirillo and Perez, 2015). Indeed, anodal tDCS of the AG also failed to affect multiplication performance despite significant BOLD activation in the retrosplenial cortex (Clemens et al., 2013). This could explain the short duration of retrieval impairments (only lasted immediately after stimulation), as well as, the low ER (5.4%) we and another rTMS study (30%) observed after left AG stimulation (Maurer et al., 2016). This reasoning might also explain why a lesion of the left AG is neither a sufficient nor a necessary condition to observe a deficit in multiplication (van Harskamp et al., 2002, 2005).

For the online calculation of the solution, stimulation of the left AG elicited robust RT slowing that lasted for 30 min in multiplication, while in subtraction RT slowing was only observed immediately after the stimulation. The impairment in the online calculation was also unexpected because arithmetic problems that require quantity manipulations were thought to be processed in the hIPS (Dehaene et al., 2003). It is therefore unclear, why left AG stimulation markedly disrupted online calculation of solution to multiplication problems. In theory, the strategy-of-choice for simple multiplication problems is retrieval. However, when retrieval fails, for instance when faced with more complex operations such as multi-digit multiplication or interference due to stimulation, a participant may adaptively use another strategy (e.g., online calculation) to produce a response. For instance, whenever direct fact retrieval for an arithmetic problem fails, bilateral intraparietal areas may be involved in semantic re-coding of the problem, recruiting magnitude information of the numbers involved (Dehaene, 1995; Klein et al., 2013b). This might have been the scenario in our participants because retrieval was impaired during and immediately after the stimulation of the left AG. However, if online calculation involves the decomposition of the arithmetic problem into smaller facts (e.g., 14 − 8 = 14 − 4 = 10 − 4 = 6), impaired retrieval of these smaller facts from verbal long-term memory will in turn negatively affect the efficiency of procedural strategy (De Smedt and Boets, 2010). Therefore, we argue that the impairment in the online calculation of answers could be secondary to the impairment in retrieval. As reflected by our imaging results, the strong activations in the cingulate, motor and frontal cortices might reflect not only the increased conflict during the fact-retrieval processes but also higher demands for controlling and coordinating multiple processing steps when a problem cannot be solved by direct retrieval (Jost et al., 2009). Additionally, the use of online calculation would be a costly strategy because this puts higher demands on verbal working memory, which might lead to slower performance in solving multiplication problems (Hecht, 2002; De Smedt and Boets, 2010). This could explain why the performance in double-digit additions that were probably solved using online calculation (more than retrieval) was also disrupted by bilateral DCS and rTMS stimulation of the AG (Roux et al., 2003; Göbel et al., 2006; Montefinese et al., 2017). On the other hand, subtraction problems solved by online calculations were not profoundly affected by the stimulation of the left AG because this strategy was thought to be carried out by the hIPS (Dehaene et al., 2003). As shown by our results, the RTs for subtraction problems solved by online calculations were not markedly prolonged by the stimulation of the left AG, as well as the vertex. In contrast, operations (double-digit addition and subtraction) that require online calculation were significantly impaired by rTMS stimulation of the left or bilateral hIPS (Göbel et al., 2006; Montefinese et al., 2017).

### RTMS Stimulation of the Left hIPS

The results from the stimulation of the left hIPS were also unexpected because we initially predicted that left hIPS stimulation would impair our participants’ ability to solve arithmetic problems that require genuine quantity manipulations such as subtraction (Dehaene et al., 2003). Instead, we did not observe any detrimental effects such as RTs slowing or increased ER in subtraction as well as in multiplication problems during and after left hIPS stimulation. Nevertheless, the effect of stimulation on RTs was strategy-dependent: retrieval was not affected whereas online calculation was improved by the stimulation in both operations. Retrieval was comparable in both sham and left hIPS stimulation conditions indicating that the left hIPS had no or only a minimal role in the retrieval of solutions from memory in subtraction and multiplication problems. Moreover, our behavioral finding was consistent with our imaging results because we did not observe significant hIPS activation during retrieval. Therefore, we argue that retrieval was not affected by the stimulation of the left hIPS because this strategy does not entirely depend on it. On the other hand, RTs for problems solved by online calculation decreased after left hIPS stimulation indicating an improvement in our participants’ ability to solve both operations using this strategy. Our imaging results also showed significant activations of the bilateral hIPS during the online calculation. This was consistent with the reported recruitment of brain areas involved in numerical quantity processing when participants were solving untrained (calculated) more than trained (mostly retrieved) subtraction and multiplication problems (Simon et al., 2002; Ischebeck et al., 2006). In contrast, our behavioral results did not corroborate the findings of previous rTMS studies that showed performance disruption in arithmetic operations (e.g., double-digit addition and subtraction) that need online calculation (Göbel et al., 2006; Montefinese et al., 2017). The performance improvement could not be due to a learning effect because it was specific for problems solved using an online calculation. Here, we may ask, why would an inhibitory rTMS stimulation paradigm applied to the left hIPS improve online calculation? For subtraction, one possible reason is that we did not stimulate and therefore inhibit the right hIPS. According to previous studies, subtraction-related areas are also predominantly localized in the right hIPS (Cohen et al., 2000; Andres et al., 2011; Maurer et al., 2016). This argument is in good accordance with the recent results from Montefinese et al. (2017) that highlighted the role of the right hIPS, as well as, the right ventral segment of IPS (vIPS) in solving complex arithmetic operations. In their study, bilateral hIPS and vIPS high frequency rTMS stimulation disrupted double-digit addition and subtraction. They argued that the stimulation disrupted online calculation because during complex arithmetic problem solving our reliance on visuospatial strategies, a suggested function of the right IPS, increases (Montefinese et al., 2017). In theory, the complexity of our subtraction problems (e.g., the requirement to conduct a “carry” procedure) may have facilitated the recruitment of the right IPS and engage visuospatial strategies as shown by the bilateral hIPS activation during the online calculation. Therefore, the recruitment of the right hIPS and the use of visuospatial strategies might have facilitated task performance because this strategy not only enhances numerosity processing and length categorization but also the processing of serial position information on the spatially oriented mental number line in mental arithmetic (Dormal et al., 2012; Knops and Willmes, 2014; Montefinese et al., 2017). Indeed, impairment not only in numerical but also in spatial bisection tasks was reported in patients with a lesion in the right parietal cortex (Zorzi et al., 2002; Cappelletti et al., 2007). Our results also showed that online calculation improvement was more robust in subtraction than multiplication problems. Here, we suggest that subtraction was less affected by inhibitory stimulation because subtraction-related areas of the cortex are known for being robust toward brain lesions or aphasia, in contrast to multiplication- or division-related cortical areas (Lampl et al., 1994; Pesenti et al., 1994; Maurer et al., 2016). Lastly, we also suggest the same arguments to explain the performance improvement in multiplication problems solved by online calculation. A study highlighted the similar role of the right IPS in multiplication by showing that single-pulse rTMS stimulation of IPS in either hemisphere (compared to control sites) led to increased RTs in addition and multiplication. They suggest that computational efficiency is not specifically dependent on left hemisphere regions and that efficiency in multiplication is dependent on the right vIPS considered to be critical for motion representation and automatization (Salillas et al., 2012).

## Conclusion

The present findings emphasized the presence of two distinct cortical networks that are modulated by the strategy and not by the arithmetic operation *per se*. For instance, we have shown that the integrity of the left AG is required in performing retrieval and online calculation strategy in multiplication, but only for the retrieval strategy in subtraction. On the other hand, the results from the stimulation of the left hIPS may indirectly suggest that the integrity of the right hIPS was sufficient to perform both operations, particularly when using the online calculation strategy. However, we would like to emphasize that great care must be taken in correlating our results with previous rTMS studies because none of them took into account the strategy used by the participants. The same principle must be applied in interpreting the correlation between our results and the findings from brain imaging studies in healthy participants, as well as, electrophysiological and neuropsychological studies in patients. This is because neuroimaging can elucidate brain areas involved in a certain task but it does not allow any causal interpretation, that is, it cannot be deduced from neuroimaging alone which areas are indeed essential for calculation. Studies done on tumor patients (mostly single-case studies) should also be interpreted cautiously since slow-growing tumors can shift the calculation-related areas and affect other parietal areas that are involved in arithmetic operations. Overall, the present findings addressed some of the disparities from previous studies. Most importantly, our findings can be a basis for developing therapeutic interventions aimed at reducing the effects of developmental dyscalculia or acquired numerical disability (Lepage and Théoret, 2010). It was already shown that increasing the excitability of the right and left parietal cortex in healthy adult participants using tDCS improved numerical ability (e.g., greater learning rates for subtraction) that lasted for 24 h up to 6 months after stimulation (Cohen Kadosh et al., 2010; Grabner et al., 2015). However, this effect was not replicated in a pilot study on two adults with developmental dyscalculia (Iuculano and Cohen Kadosh, 2014). Therefore, further investigations are warranted particularly those that focus on the strategy that these individuals are often using when solving numerical problems.

## Data Availability Statement

The datasets generated for this study are available on request to the corresponding author.

## Ethics Statement

The studies involving human participants were reviewed and approved by Ethics committee of the Medical University Graz. The patients/participants provided their written informed consent to participate in this study.

## Author Contributions

SF, CK, EG, GC, and AI designed the study. SF, SP, MJ, and KZ conducted fMRI experiments. SF, MC, SP, MH, KM, and KZ conducted the rTMS experiments. SF and AI analyzed the data and wrote the manuscript. SF, MC, SP, MJ, KZ, MH, KM, EG, CK, GC, and AI reviewed and approved the final version of the manuscript.

## Conflict of Interest

The authors declare that the research was conducted in the absence of any commercial or financial relationships that could be construed as a potential conflict of interest.
