# Supplementary material for: Dissociating Arithmetic Operations in the Parietal Cortex Using 1 Hz Repetitive Transcranial Magnetic Stimulation: The Importance of Strategy Use
Source: Front Hum Neurosci. 2020 Jul 16;14:271. doi: 10.3389/fnhum.2020.00271 (PMC7378795; doi:10.3389/fnhum.2020.00271)
Supplement: Supplementary file 1 [file Table_1.docx]

Dissociating arithmetic operations in the parietal cortex using 1Hz repetitive transcranial magnetic stimulation: The importance of strategy use

| **Parameters** | **AIC** | **Δᵢ (AIC)** | Relative model likelihoods | ***w*ᵢ (AIC)** |
| --- | --- | --- | --- | --- |
| Random intercept | 260167.359 | 1245.743 | 0.000 | 0.000 |
| + stimulation site | 260143.655 | 1222.039 | 0.000 | 0.000 |
| + operation | 259999.694 | 1078.078 | 0.000 | 0.000 |
| + strategy | 259642.985 | 721.369 | 0.000 | 0.000 |
| + time | 259593.682 | 672.066 | 0.000 | 0.000 |
| + stimulation site x operation | 259564.077 | 642.461 | 0.000 | 0.000 |
| + stimulation site x strategy | 259538.047 | 616.431 | 0.000 | 0.000 |
| + stimulation site x time | 259420.804 | 499.188 | 0.000 | 0.000 |
| + operation x strategy | 259356.997 | 435.381 | 0.000 | 0.000 |
| + operation x time | 259320.932 | 399.316 | 0.000 | 0.000 |
| + strategy x time | 259275.478 | 353.862 | 0.000 | 0.000 |
| + stimulation site x operation x strategy | 259253.582 | 331.966 | 0.000 | 0.000 |
| + stimulation site x operation x time | 259165.768 | 244.152 | 0.000 | 0.000 |
| + stimulation site x strategy x time | 259072.244 | 150.628 | 0.000 | 0.000 |
| + operation x strategy x time | 259028.702 | 107.086 | 0.000 | 0.000 |
| + stimulation site x operation x strategy x time | 258921.616 | 0 | 1.000 | 1.000 |
|  |  |  |  |  |
|  |  |  | Sum = 1.000 |  |

Supplementary Table 1. The Akaike Information Criterion (AIC) and Akaike weights of the competing models. The differences (Δᵢ (AIC)) in AIC with respect to the AIC value of the best candidate model (model with the lowest AIC value) was first calculated. From Δᵢ (AIC), we determined the relative model likelihoods. The relative model likelihoods were then normalized by dividing each value to the sum of the likelihoods of all models to determine the Akaike weight (wᵢ (AIC)) of each model. AIC = Akaike Information Criterion value.
