# Supplementary material for: Dissociating Arithmetic Operations in the Parietal Cortex Using 1 Hz Repetitive Transcranial Magnetic Stimulation: The Importance of Strategy Use
Source: Front Hum Neurosci. 2020 Jul 16;14:271. doi: 10.3389/fnhum.2020.00271 (PMC7378795; doi:10.3389/fnhum.2020.00271)
Supplement: Supplementary file 2 [file Table_2.docx]

Dissociating arithmetic operations in the parietal cortex using 1Hz repetitive transcranial magnetic stimulation: The importance of strategy use

|  | **Numerator df** | **Denominator df** | ***F*-value** | ***p-*value** | **Cohen’s D** |
| --- | --- | --- | --- | --- | --- |
|  |  |  |  |  |  |
| Time | 4 | 358.80 | 3.6 | .006* | .420 |
| Operation | 1 | 359.63 | .083 | .774 | .072 |
| Stimulation site | 2 | 360.17 | 1.16 | .314 | .248 |
| Time x operation | 4 | 358.37 | .164 | .957 | .071 |
| Time x stimulation site | 8 | 358.62 | .424 | .907 | .125 |
| Operation x stimulation site | 2 | 358.88 | 1.35 | .260 | .267 |
| Time x operation x stimulation site | 8 | 358.35 | .435 | .900 | .162 |

Table 2. Results of the linear mixed model (LMM) performed on the error rates from the rTMS experiment. For the LMM (random intercept model), each participant was treated as a random factor. The within-subjects factor stimulation site (hIPS, AG, and vertex), operation (multiplication and subtraction), and time (before, during, and 0, 30 and 60 min after stimulation) were treated as fixed factors. Asterisks indicate significant results (*p* < 0.05) df, Degrees of freedom.
